# Research on the expected utility of the nudge-based intervention strategy for healthy eating behavior in online catering

**DOI:** 10.3389/fpubh.2025.1644713

**Published:** 2025-10-28

**Authors:** Xiaoting Dai, Linhai Wu

**Affiliations:** ^1^Jiangsu Provincial Laboratory of Food Safety and National Strategic Governance, Jiangnan University, Wuxi, China; ^2^School of Business, Jiangnan University, Wuxi, China

**Keywords:** online food, nudging strategy, eating behavior, healthy eating, dietary intervention

## Abstract

**Introduction:**

While the rise of the new consumption format of “Internet + Food” has changed the global food environment, it has also caused people to worry about the health risks brought about by the unreasonable dietary structure of online catering foods. Under the online catering consumption model based on electronic transactions, compared with traditional tough intervention policies, the nudging strategy can make full use of the advantages of information technology and effectively promote consumers’ choice of healthy diet to interfere with the dietary behavior of online catering consumers.

**Methods:**

The objective of this study is to design a choice environment on online platforms through nudge strategies, so as to reduce the cognitive resistance and inertia of consumers in decision-making. By means of guidance rather than coercion, it enables consumers to make healthy choices more easily and proactively, thereby promoting the occurrence of healthy eating behaviors invisibly. Based on the subdividing of respondents based on the characteristics of consumer groups, we explored the different expected intervention effects of providing decision information, changing decision structure, and providing decision-making assistance-type nudging strategies on consumers with different characteristics. This paper divides the nudging strategies into three categories: providing decision information type, changing decision structure type, and providing decision assistance type. The subjects of this paper must be consumers who have their own experience of eating online food and beverages. A total of 44,050 valid samples were collected.

**Results:**

The results found that nudging strategies have a positive impact on healthy eating behaviors; specifically, women, lower education, lower income consumers, and chronic patients are more susceptible to the healthy diet-nudging strategies.

**Discussion:**

Compared with providing decision-making information type and changing decision-making structure type by constructing multinomial logistic regression models, the intervention effect of providing decision-making auxiliary nudging strategies is slightly insufficient. Changing the structural nudge strategy of decision-making is more influential among high-age, low-education and low-income consumer groups.

## 1 Introduction

With the rise of the new economic form of “Internet +,” the online catering industry based on electronic transactions has developed rapidly, which has inherently triggered disruptive changes in the production, processing, operation and consumption methods of catering food worldwide. According to the global authoritative statistical database Statista (2024), the global online catering market size will reach US$436.5 billion in 2024, of which, China’s online catering market size will reach US$182.9 billion, accounting for about 41.9% of the global total, and China’s online catering user penetration rate will reach 54.5% (1). It is estimated that the global market size will maintain an average annual growth rate of around 4.6% from 2024 to 2028. The rise of the online catering industry has greatly changed the food environment (Food environment), and it is expected that people’s dependence on online catering food will further increase in the future. However, compared with traditional family cooking or dine-in dietary patterns, the rationality of dietary structure of online catering foods is questioned (2).

Data released by the World Health Organization (3) shows that approximately 5% of global deaths each year, or about 2.8 million people, can be attributed to obesity caused by unhealthy diets. Unhealthy diets are primarily characterized by low intake of fruits and vegetables and excessive consumption of foods high in salt, saturated fat, trans fats, and added sugars. In 2020, data from the WHO showed that among the top ten leading causes of death globally in 2019, the first seven were all related to chronic diseases, accounting for 74% of total global mortality (4). This suggests that roughly 41 million people worldwide die each year due to chronic diseases. Larentis et al. (5) research indicates that chronic diseases and their associated risk factors have been on a surge. The fact that during the global outbreak of COVID-19, patients with chronic diseases had a higher probability of dying from the virus compared to healthy individuals highlights the critical importance of a healthy immune system in combating viruses and reducing mortality rates (6). Chronic diseases have become a major public health issue severely threatening human health globally (7). However, studies have confirmed that unreasonable dietary behaviors and poor dietary structure are key factors in the development of chronic diseases (8). Scientific and healthy eating habits, along with a balanced diet, can help reduce the prevalence of chronic diseases and the continuous rise in premature mortality (9). There is a global emphasis on improving dietary patterns and optimizing dietary structures (10).

The concept of nudge was developed by Richard H. Thaler was first proposed in 2008, which is defined as using slight and more “implicit” intervention strategies to guide individual behavior to change in the expected direction on the basis of fully considering individual irrational behavior, thus leading to nudging strategies, integrating psychology, behavioral economics, etc. into public policy formulation, which focuses on behavior change and advocates “libertarian Paternalism” management, which neither forces or restricts individuals to make choices nor allows individuals to be free (11, 12).

Scholars have studied nudging strategies in business, government, health care, public health, and many other fields. In public health, "nudging” has been used to encourage choices to improve health behaviors and outcomes, including screening tests, medication compliance, physical activity, dietary behaviors, etc. (13), especially in the intervention of healthy eating behavior, foreign scholars have carried out a lot of exploration and research on nudging strategies, and unanimously believe that nudging strategies have significant effects on promoting consumers ‘nutritional and healthy food choices and paying attention to changes in food selection environment. The research sites include restaurants, convenience stores, retail food environment, farmers’ markets, etc. (9). For example, numerous studies have shown that nudging strategies can promote healthy dietary consumption (14) and sustainable food choices (15). It was found that putting traffic light labels on workplaces and hospital buffets significantly increased sales of healthy foods and increased healthy food choices (16, 17). Payne and Niculescu (18) placed fruits and vegetables at the end of grocery checkout aisles and asked cashiers to suggest purchases to customers, a combination that led to a significant increase in fruit and vegetable purchases. Otto et al. (19) using local norms specific to the food consumption environment to nudge consumers to make healthier choices (e.g., "In this store in this part of the city, people will order an average of 250 calories”) resulted in a significant reduction in sales of higher-calorie foods. Cesareo et al. (20) by investigating the impact of multiple nudging interventions (i.e., increased convenience, disclosure of “So Good” labels, drawing attention with posters, etc.) on college students ‘diets, it was found that nudging strategies successfully promoted healthy menu choices.

Based on the rapid development of the online catering industry and China has the world’s largest online catering market (1), combined with the objective reality of the unreasonable dietary structure of online catering foods (21), and considering that unhealthy diets are very likely to cause chronic diseases such as obesity, hypertension, hyperlipidemia, diabetes, etc., it has become an important risk factor affecting the health of residents around the world (22). It can be seen that consumers’ online catering dietary choices have become particularly important, and it is urgent to conduct research focusing on interfering with consumers’ healthy dietary behaviors. Based on this, this paper fully considers the characteristics of online catering consumer groups, aiming to formulate different nudging strategies based on the common characteristics of different subdivided populations. By collecting survey data on the willingness of online catering consumers in China to accept the nudging strategies, the research method of multiple logistic regression model is adopted to explore the different expected intervention effects of three types of nudging strategies, such as providing decision information, changing decision structure, and providing decision assistance, on different types of consumers.

## 2 Literature review

As the popularity of online food delivery increases, scholars have begun to pay attention to health issues caused by online food consumption. Online food is often associated with high-calorie content, low nutritional value, excessive added sugars, high fat, and high salt levels—characteristics that are detrimental to health (23). These dietary features have been confirmed as key risk factors for obesity, high cholesterol, diabetes, and hypertension (24). Studies by Dana et al. (25) indicate that the unbalanced dietary structure provided by online food delivery is a critical factor leading to overweight and obesity. Horta et al. (26) found that long-term consumption of monotonous online food can lead to chronic diseases such as obesity, hypertension, and diabetes (u). Du et al. (27) further confirmed that the dietary and nutritional structure of off-premises food is severely imbalanced, and frequent consumption of off-premises food is significantly associated with an increased risk of all-cause mortality, with a rate as high as 49%. And this trend is spreading among young people, especially those at a younger age, potentially becoming a significant global social issue (24). The Centers for Disease Control and Prevention in the United States (54) emphasized that optimizing daily dietary intake may help slow or even reduce the continuous rise in chronic disease prevalence and premature mortality, which has sparked reflection and attention from both government and academia on interventions to promote healthy eating behaviors. However, there are currently no studies specifically addressing the correlation between health behavior interventions and online food consumers.

Health behaviors refer to any form of observable actions or response patterns actively adopted by individuals or groups to maintain, promote, or restore health, as well as prevent diseases. These behaviors are based on existing scientific evidence and have been proven to have positive and measurable benefits for physical and mental health (28). Over the years, public health organizations, governments, consumers and other interest groups have promoted various interventions to promote healthy food choices. Traditional policy instruments to promote healthy diets have focused on government intervention through laws, regulations and economic measures such as taxes and subsidies, Britain, the United States and other countries have even adopted tough policies such as taxation and sales restrictions on unhealthy foods to reduce people’s consumption of unhealthy foods. However, the effect of high-cost investment is very limited, and even causes people to fight for freedom of diet. It also includes promoting health science popularization, encouraging enterprises to produce and sell healthy food, and improving the awareness rate of residents ‘nutrition labels. However, it does not obviously show the intervention effect on residents’ choice of healthy food (14). A slight, more “implicit” nudge strategy than traditional policies can fully understand the irrational side of an individual’s food choices, and it does not necessarily need to provide explicit information about food, as long as the cue or stimulus causes people to consciously or subconsciously make healthy choices (29, 30), the multiple advantages of boost strategy are widely concerned and recognized by academia and government (31), scholars concluded that it has the advantages of less resistance to consumers, lower implementation cost and higher flexibility (32).

Nudging strategies are sophisticated intervention tools rooted in the wisdom of behavioral science. They profoundly recognize the irrational nature of human decision-making. By redesigning choice architectures and systematically leveraging cognitive biases and psychological mechanisms (such as the default effect, social norms, and loss aversion), they gently guide people’s automatic, fast thinking. This effectively reduces barriers to adopting healthy lifestyles while preserving individuals’ freedom of choice, ultimately contributing to the promotion of public health and individual well-being. Though not a panacea, they offer a highly cost-effective and ethically appealing theoretical path and practical tool for addressing complex challenges in health behaviors (12, 33). Scholars both domestically and internationally have constructed different framework systems for the promotion strategies in public policy from various perspectives (34). In the literature on the framework of health eating behavior promotion strategies, the establishment of frameworks is mainly based on three methods: one method is to construct a framework according to the process by which promotion strategies influence cognition (cognitive processes), for example, Wilson et al. (30) proposed that the framework of health eating promotion strategies should focus primarily on three categories: activation-type promotion (Priming nudges), salience-type promotion (Salience nudges), and combined activation and salience promotion (Priming and Salience nudges combined). The second method is to categorize specific promotion strategies based on their concrete form (35). For example, Cadario and Chandon (36) categorized the strategy framework into three dimensions—cognitive orientation (Cognitively oriented), affective orientation (Affectively oriented), and behavioral orientation (Behaviorally oriented)—based on the different purposes of health diet promotion strategies. Another approach divides strategies based on their impact mechanisms on decision-making systems (11), such as Munscher et al. (37), who established a strategy framework distinct from previous research systems, categorizing promotional strategies into three major types—providing decision information (decision information), altering decision structure (decision structure), and providing decision support (decision assistance).

In summary, scholars at home and abroad have carried out a lot of academic research and practical exploration on nudging strategies in terms of intervening healthy eating behaviors, which are rich in theory and practice. At the same time, scholars have also made great efforts to construct a strategic framework for intervening in public healthy eating behaviors. However, in view of the rapid growth of public demand for online catering food, the effectiveness of healthy diet intervention strategies from the perspective of the online catering industry has not been studied, and the effectiveness of the nudging strategies for different types of consumers has been analyzed in a targeted manner, and it has been seldom found that empirical studies have compared and analyzed different strategies in the nudge strategy framework system. As Bang et al. (38) elaborate, the same nudge strategy may have different effects on different populations with different characteristics and for different interventions. Especially in China, which has the largest scale of online catering in the world, similar studies are scarce. Based on the shortcomings of previous studies, this paper tries to fill the gaps in the above literature, and its main efforts and contributions are as follows: based on the Chinese context, based on the objective reality of the unreasonable dietary structure of online catering food at this stage, the sample data of consumers’ willingness to accept the booster strategy are collected through a questionnaire survey, and the expected intervention effect of the booster strategy on consumers’ healthy eating behavior is studied by using multiple logistic regression models. The different impacts of the three booster strategies of changing the decision-making structure and providing decision-making assistance on consumers with different characteristics.

## 3 Research methodology

### 3.1 Research method and variable setting

**Figure 1:**
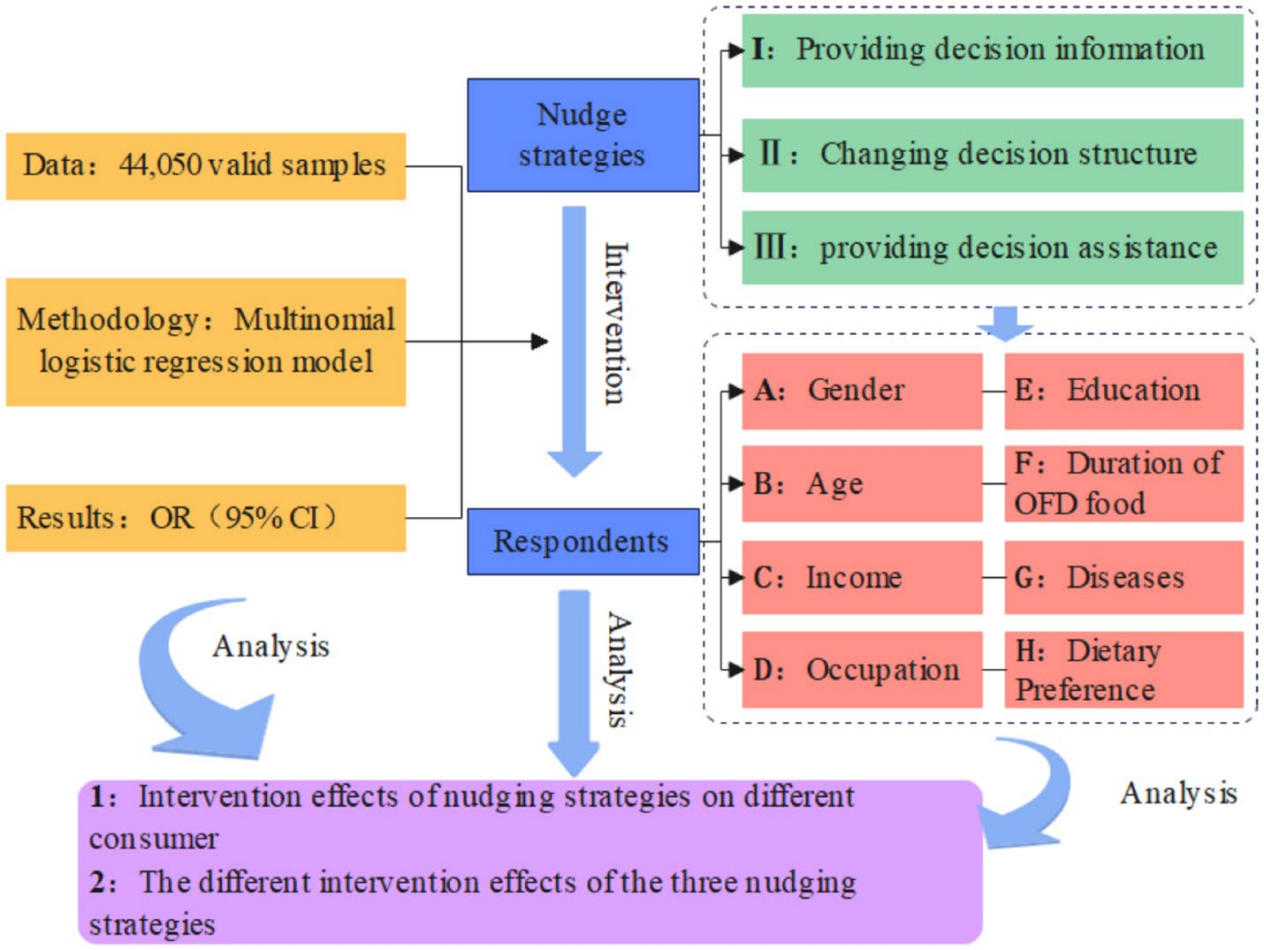
Diagram of the research framework.

This paper studies the expected effectiveness of intervention strategies that promote healthy eating behaviors. At the same time, different types of consumers evaluate the feedback differences in different categories of nudging strategies, and constructs a research framework diagram as shown in Figure 1. This paper divides the nudging strategies into three categories: providing decision information type (I), changing decision structure type (II), and providing decision assistance type (III). According to Bang et al. (38), facing people with different characteristics and for different intervention content, the same nudge strategy may have different effects. Therefore, this article classifies respondents according to consumer group characteristics. The basis for setting group characteristics is to learn from the research of Loibl et al. (39), and include individual characteristics (gender, age, education, occupation), socio-economic characteristics (annual income), lifestyle and attitude towards life (online catering food history, dietary preference^1^) in the classification criteria. In addition, this paper designs a feature that distinguishes it from previous studies, namely whether the respondents suffer from chronic diseases (specifically divided into obesity, hypertension, diabetes, hyperlipidemia, gout, and tumors). The reason is that based on the study results of unreasonable dietary behaviors and dietary structures that are key factors in causing chronic diseases (8), the importance of healthy dietary interventions to patients with chronic diseases is self-evident. If targeted nudging strategies can be formulated based on this characteristic, it may help reduce the prevalence of chronic diseases and even the continuous increase in premature mortality (9). Therefore, this article grouped respondents based on the above eight consumer group characteristics, as shown in Table 1.

**Table 1:** Classification of consumer group characteristics.

Group	Characteristics	Categories
A	Gender	Male; Female
B	Age (year)	18–30, 31–45, 46–60, 61–70
C	Personal annual income (yuan)	<30,000; 30,000–50,000; 50,000–100,000; 100,000–150,000; 150,000–200,000; >200,000
D	Occupation	Civil servant; Company employee; Public institution employee; Farmer; Self-employed; Unemployed; Retired; Student or graduate student; Others
E	Education	Junior high school or lower; High school; Junior college; Bachelor’s degree; Master’s degree or higher
F	Duration of OFD food consumption	hardly ever buy; 0–1 year; 1–3 years; 3–5 years; more than 5 years
G	Diseases	Obese; Hypertension; Diabetes; Hyperlipidemia; Gout; Tumors
H	Dietary preference	Rice meals; Pizzas and hamburgers; Fast hot pot; Crayfish and barbecue; Fried chicken and fried skewers; Congee and gruel; Noodle set meal; Japanese and Korean cuisine; Western cuisine; Salad; Coffee; Fruits; Milk tea and desserts

In this paper, eight groups of categories are analyzed for comparison. Therefore, in each group, there is a control group and *n* (n ≥ 1) observation groups, respectively. For example, in the group classified according to sex, the control group is male respondents, and the observation group is female respondents. Drawing on Du et al. (27), this paper constructs a multinomial logistic regression model to estimate the possible impact of different categories of nudging strategies on different types of respondents after covariate adjustment. Explanatory variables in the model are different types of consumers, and outcome variables are respondents ‘feedback willingness to three categories of nudging strategies, i.e., self-assessment of whether they will make healthy eating choices after receiving information about nudging strategies. Multinomial logistic regression models included residence, marital status, family size, and presence of children under 12 as covariates (39).

### 3.2 Research subjects, sample determination and hypotheses

Drawing on Munscher et al. (37), this paper designed a prospective effectiveness study of a boost-based intervention strategy for healthy eating behavior in online dining. The questionnaire is divided into two parts: the first part contains the basic information of the consumers surveyed (hereinafter referred to as respondents), including gender, age, education level, marital status, personal and family income, occupation, frequency of eating online meals, whether suffering from chronic non-communicable diseases, etc.; the second part is the respondents ‘willingness to accept the nudge strategy, that is, whether they will make healthy eating choices under the influence of the nudge strategy when choosing food. According to Munscher et al.’s (37) nudge strategy framework, the nudging strategies in the questionnaire are divided into three categories: providing decision information (I), changing decision structure (II) and providing decision assistance (III). The nudge strategy of providing decision information is reflected in changing the presentation form of existing information, so that consumers can receive and utilize useful information more effectively. In the questionnaire, it is expressed as asking respondents “When ordering takeout, if food nutrition prompt labels appear on the website, Will it help you choose healthy food?” (36). The modified decision structure motivating strategy is to increase the likelihood that consumers will actively or unconsciously choose healthy foods by improving the health attributes of the selected food options themselves. It is reflected in the questionnaire asking respondents “When ordering takeout, if the healthier foods are ranked first, it will make it easier for you to buy them” (40). Providing a decision-supporting nudge strategy means that consumers can make healthy eating choices by implementing reminder measures before making decisions, which is reflected in the questionnaire asking respondents whether “When ordering takeout settlement, if the reminder” You do not seem to eat very healthy today “appears on the website, will you go back and choose again” (41) (see Supplementary material).

The research hypotheses of this paper are as follows, *H1:* Nudging strategies have a positive impact on the healthy eating behaviors of respondents with different profiles, and the impact varies based on eight different basic characteristics; *H2:* Different types of nudging strategies have varying degrees of impact on respondents’ healthy eating behaviors.

### 3.3 Data collection and validity test

A small-scale pilot study was initially conducted to ensure the accuracy and acceptability of the questionnaire items. Subsequent to this, a large-scale formal questionnaire survey was implemented. Specifically, an online survey link was created on a website; this link was then shared on social media platforms for wider dissemination, with the aim of improving the response rate. Prior to initiating the questionnaire, i.e., when respondents accessed the first page of the survey, informed consent was obtained. Respondents were only able to proceed with completing the questionnaire upon providing their consent to participate. The survey was administered anonymously, ensuring that the personal privacy of all respondents remained protected and undisclosed.

Except for a few multiple-choice questions, most of the survey questions are multiple-choice questions, and uncertainty options are set in the answers of each question item to ensure that the real physical condition of the respondents’ consumption and self-assessment is known. The important concepts involved in the questionnaire (e.g., “nutrition label,” “tumor” type, “blood lipid” normal range values, etc.) are specifically explained in the questionnaire. The samples were collected from May 01 to June 30, 2023. The subjects of this paper must be consumers who have their own experience of eating online food and beverages. After removing the samples that did not meet the basic criteria of the respondents, a total of 44,050 valid samples were collected.

This paper verifies the overall validity of model coefficients through the Omnibus test to examine whether the model as a whole has explanatory power. As shown in Table 2, the Omnibus test results of the three models (models of the impact of three types of nudging strategies on respondents) are presented by Chi-square value, Degrees of Freedom (df), and significance (*p* value). It can be found that all Chi-square values are relatively large, indicating that after incorporating all independent variables, the model fitting effect is good, that is, the overall “contribution degree” of the coefficients is higher. The *p* value < 0.05 indicates that the model as a whole is valid and statistically significant.

**Table 2:** Omnibus test of model coefficients.

Models	Chi-square value	Degrees of freedom, df	Significance, *p* value
Model 1 (providing decision information)	1998.689	52	0.000
Model 2 (changing decision structure)	2986.787	52	0.000
Model 3 (providing decision assistance)	2977.354	43	0.000

## 4 Results and analysis

### 4.1 Statistical characteristics of the sample

Table 3 shows the demographics of the respondents, 49.33% of the respondents are male, and 70.72% of the respondents live in cities of different sizes, which is consistent with the fact that the consumption pattern of online catering in China is more common in cities than in rural areas (25). More than 94% of the respondents have a high school degree or above, 60.38% of the respondents are students, employees of enterprises and institutions, and 60.37% of the respondents do not have children under the age of 12 at home, which can indirectly indicate that young people are the main group of food and beverage consumers, which is consistent with the conclusion that young people are the main consumers of online food and beverage in the world (42, 43). At the same time, 70.41% of the respondents have an annual personal income of less than 100,000 yuan, belonging to the middle-income group and below. 68.16% of the respondents have maintained the habit of eating online catering food for more than 1 year, and even 14.78% of the respondents have maintained this habit for more than 5 years. In addition, the most popular type of meal is the meal set, which 35.85% of the respondents regularly buy. According to the BMI recommended by the Working Group on Obesity in China ≥28 kg/m^2^ as obesity, the questionnaire results showed that 11.27% of the respondents were obese, and 11.27, 6.70, 12.00, 5.04 and 2.91% of the respondents reported that they had five types of chronic non-communicable diseases: hypertension, diabetes, hyperlipidemia, gout and tumor diseases. At the same time, 54.53, 54.04 and 38.57% of the respondents believed that their eating behavior would be affected by the I, II, III nudge strategy, respectively.

**Table 3:** Demographics of respondents.

Group	Sample size (*n*)	Proportion (%)
Gender		
Male	21,728	49.33
Female	22,322	50.67
Age (year)		
18–30	10,982	24.93
31–45	14,932	33.90
46–60	9,566	21.72
61–70	8,570	19.45
Marital status		
Singlehood	13,554	30.77
Married	30,496	69.23
Place of residence		
Cities and towns	31,152	70.72
Countryside	12,898	29.28
The number of family members (persons)		
1	870	1.98
2	9,742	22.12
3	20,108	45.65
4	8,468	19.22
≥5	4,862	11.03
Presence of children under the age of 12 years in the household		
Yes	17,458	39.63
No	26,592	60.37
Education		
Junior high school or lower	2,400	5.45
High school	10,292	23.36
Junior college	11,572	26.27
Bachelor’s degree	15,242	34.60
Master’s degree or higher	4,544	10.32
Personal annual income (yuan)		
<30,000	5,394	12.25
30,000–50,000	8,722	19.80
50,001–100,000	16,778	38.09
100,001–150,000	7,738	17.57
150,001-200,000	3,240	7.36
>200,000	2,178	4.93
Occupation		
Civil servant	3,454	7.84
Company employee	16,470	37.39
Public institution employee	6,232	14.15
Farmer	3,314	7.52
Self-employed/unemployed/retired	7,316	16.61
Student/graduate student	3,896	8.84
Others	3,368	7.65
Duration of OFD food consumption		
Hardly ever buy	4,668	10.60
<1 year	9,356	21.24
1–3 years	9,610	21.82
3–5 years	13,902	31.56
>5 years	6,514	14.78
Diseases		
Obese	1,416	3.21
Hypertension	4,966	11.27
Diabetes	2,952	6.70
Hyperlipidemia	5,284	12.00
Gout	2,220	5.04
Tumors	1,282	2.91
Other cases	25,930	58.87
Acceptance willingness of providing decision information (I)		
Accept	24,020	54.53
Not accept	9,554	21.69
Not sure	10,476	23.78
Acceptance willingness of changing decision structure (II)		
Accept	23,806	54.04
Not accept	11,420	25.93
Not sure	8,824	20.03
Acceptance willingness of providing decision assistance (III)		
Accept	16,990	38.57
Not accept	10,378	23.56
Not sure	16,682	37.87

### 4.2 Analysis of model results

SPSS software was used to process the questionnaire data. Odds ratios (ORs) and 95% confidence intervals (CIs) were used to explain the possible feedback of different types of consumers on the three categories of nudging strategies. Odds ratios represent the risk multiples for future occurrence of outcome variables in the observation group compared to the control group, i.e., the impact of the nudge strategy, and a 95% confidence interval means that at a 95% confidence level, this estimation interval will include the true odds ratio value. This paper analyzes eight groups of respondents classified according to different basic characteristics.

#### 4.2.1 Discuss according to the different basic characteristics of the respondents

Table 4 summarizes the possible impacts of different categories of nudging strategies on different types of respondents. It can be found that compared with male respondents, female respondents have a higher willingness to accept nudging strategies. The data show that women are 1.01 (95%CI 0.96 to 1.061), 1.029 (95%CI 0.983 to 1.078), and 1.019 times (95%CI 0.921 to 1.102) of male respondents, that is, they are more susceptible to nudging strategies to make healthy catering food choices. The basic characteristic of “age” also significantly affects the intervention effect of nudging strategies on respondents. Respondents aged 18–30 are more likely to accept type I and type III nudging strategies than those aged 30. For example, respondents aged 31–45 are 0.686 times more likely to be affected by type I nudging strategies than consumers aged 18–30 (95%CI 0.635 to 0.741). In particular, the older respondents are less likely to be willing to accept Type I, that is, they are less willing to choose healthy catering foods by analyzing the information on the food nutritional content tip label. For example, respondents aged 61–70 are only 0.572 times more likely to be affected by Type I nudging strategies than those aged 18–30 (95%CI 0.521 to 0.628). Mazza et al. (44) studied the utility of nutritional information labels. They found that traffic light labels containing calorie information affect consumers’ purchasing strength for high-calorie foods. Based on this research results, this paper further found that the lower the age respondents are more susceptible to the promotion strategies for providing decision-making information (44).

**Table 4:** The expected intervention effects of three types of nudging strategies on different consumers (A–F).

Group	Variables	Categories	Providing decision information (I) OR (95% CI)	Changing decision structure (II) OR (95% CI)	Providing decision assistance (III) OR (95% CI)
A	Gender	Male (referent)	1 (referent)	1 (referent)	1 (referent)
		Female	1.01 (0.96, 1.061)	1.029 (0.983, 1.078)	1.019 (0.921, 1.102)
B	Age	18–30 (referent)	1 (referent)	1 (referent)	1 (referent)
		31–45	0.686 (0.635, 0.741)	1.025 (0.955, 1.101)	0.852 (0.787, 0.922)
		46–60	0.6 (0.549, 0.655)	0.963 (0.886, 1.046)	0.752 (0.687, 0.824)
		61–70	0.572 (0.521, 0.628)	1.153 (1.056, 1.258)	0.891 (0.809, 0.981)
C	Personal annual income (yuan)	<30,000 (referent)	1 (referent)	1 (referent)	1 (referent)
		30,000–50,000	1.190 (1.158, 1.273)	1.114 (1.023, 1.213)	1 (0.91, 1.099)
		50,001–100,000	1.177 (1.081, 1.281)	0.992 (0.917, 1.072)	1.175 (1.078, 1.281)
		100,001–150,000	0.935 (0.848, 1.03)	0.796 (0.728, 0.871)	1.015 (0.92, 1.121)
		150.001–200,000	1.172 (0.744, 0.954)	0.76 (0.678, 0.852)	1.099 (0.971, 1.243)
		>200,000	1.189 (1.034, 1.368)	0.695 (0.607, 0.796)	0.939 (0.816, 1.08)
D	Occupation	Civil servant (referent)	1 (referent)	1 (referent)	1 (referent)
		Company employee	0.892 (0.81, 0.984)	0.89 (0.814, 0.973)	0.969 (0.875, 1.074)
		Farmer	1.151 (1.017, 1.302)	1.199 (1.071, 1.343)	1.12 (0.982, 1.278)
		Public institution employee	0.838 (0.749, 0.937)	0.905 (0.816, 1.003)	0.908 (0.809, 1.019)
		Self-employed	0.984 (0.874, 1.108)	0.972 (0.87, 1.085)	1.108 (0.981, 1.252)
		Retired	1.001 (0.762, 1.313)	0.688 (0.523, 0.905)	0.797 (0.601, 1.058)
		Unemployed	0.932 (0.815, 1.066)	0.841 (0.743, 0.953)	1.358 (1.179, 1.565)
		Student/graduate student	1.182 (1.097, 1.378)	0.895 (0.793, 1.01)	1.143 (1.049, 1.255)
		Others	0.812 (0.714, 0.924)	0.665 (0.587, 0.753)	0.894 (0.783, 1.022)
E	Education	Junior high school or lower (referent)	1 (referent)	1 (referent)	1 (referent)
		High school	1.181 (1.049, 1.33)	0.899 (0.804, 1.005)	0.967 (0.858, 1.09)
		Junior college	1.103 (0.981, 1.241)	0.915 (0.819, 1.022)	0.964 (0.857, 1.085)
		Bachelor’s degree	0.772 (0.686, 0.87)	0.717 (0.642, 0.801)	0.732 (0.651, 0.824)
		Master’s degree or higher	0.834 (0.725, 0.961)	0.718 (0.63, 0.819)	0.611 (0.53, 0.704)
F	Duration of OFD food consumption	Hardly ever buy (referent)	1 (referent)	1 (referent)	1 (referent)
		<1 year	1.758 (1.577, 1.961)	2.118 (1.902, 2.358)	1.678 (1.512, 1.862)
		1–3 years	2.015 (1.808, 2.245)	1.952 (1.754, 2.173)	1.664 (1.519, 1.824)
		3–5 years	1.721 (1.549, 1.911)	1.865 (1.681, 2.07)	1.496 (1.234, 1.675)
		>5 years	1.634 (1.454, 1.835)	1.838 (1.641, 2.06)	1.355 (1.177, 1.553)

Model: The respondent’s place of residence, marital status, family population, and whether there are children under 12 years old in the family were used as covariates to evaluate the expected intervention effect of the nudge strategy on the respondents.

Compared with civil servants, respondents who are farmers were more likely to accept the nudge strategy than civil servants, and they were 1.151 (95% CI 1.017 to 1.302), 1.199 (95% CI 1.071 to 1.343), and 1.12 times (95% CI 0.982 to 1.278) of the respondents who were civil servants (Table 4). Similarly, students were more receptive to type I and type III nudging strategies, and were 1.182 (95% CI 1.097 to 1.378) and 1.143 times more likely to be affected than civil servants (95% CI 1.049 to 1.255), respectively, which is consistent with the effect of the “age” characteristic, as most of the students are concentrated in the 18–30 age range. Compared with those who were civil servants, corporate employees, freelancers, retirees, unemployed, or other respondents had more negative feedback on the booster strategy, for example, unemployed respondents were only 0.841 times more likely to be affected by the type II booster strategy than civil servants (95% CI 0.743 to 0.953).

Respondents with different educational backgrounds also had different feedback on nudging strategies. The results showed that respondents with bachelor degree or above were less willing to accept nudging strategies than those with junior high school or below. That is, respondents with bachelor degree or above were less likely to be affected by nudging strategies. For example, respondents with graduate degree or above were 0.611 times more likely to be affected by type III boosting strategies than those with junior high school or below (95% CI 0.530 to 0.704). The reason may be that education affects basic knowledge about nutrition and healthy eating. The more educated respondents are, the more they may understand and value nutrition and healthy eating, as well as the adverse effects of unhealthy eating on their health. Therefore, the impact of boosting strategies on such respondents is relatively small compared to those with lower education.

The feature of “eating history” also significantly affects the intervention effect of the boosting strategy on the respondents. It can be found from Table 4 that compared with the respondents who have not formed the habit of purchasing online meals, the respondents with different levels of eating history are more likely to be affected by the boosting strategy, and the respondents with 0–3 years of eating history are most likely to be affected by the intervention of the boosting strategy. For example, the respondents with 0–1 years of eating history are affected by I, II, The likelihood of the impact of type III boosting strategies was 1.758 (95% CI 1.577 to 1.961), 2.118 (95% CI 1.902 to 2.358), and 1.678 (95% CI 1.512 to 1.862) times higher than that of respondents who did not form habitual purchases. 1–3 Respondents with annual consumption history were 2.015 (95% CI 1.808 to 2.245), 1.952 (95% CI 1.754 to 2.173), and 1.664 (95% CI 1.519 to 1.824) times more likely to be influenced by type I, II, and III boosting strategies, respectively, than those without habitual buying. The results show that consumers who habitually purchase online food have the intention to change their dietary choices through boosting strategies, but as the duration of this habit becomes longer, consumers may gradually form a fixed dietary pattern, and the willingness to change their dietary choices will gradually weaken, and the intervention effect of boosting strategies will also weaken.

This paper also analyzed the expected intervention effects of boosting strategies for respondents with different chronic diseases (see Table 5). The results showed that compared with respondents who self-evaluated themselves without chronic diseases (control group), respondents with obesity and hypertension were less likely to be affected by boosting strategies. For example, the ratio of intervention effects of obesity and hypertension patients with type I boosting strategies was 0.834 (95%CI 0.711 to 0.980) and 0.565 times (95%CI 0.512 to 0.623) in the control group, respectively. However, respondents with diabetes, gout, and tumor chronic diseases are more likely to be affected by the intervention of boosting strategies than those without chronic diseases. Previous studies have shown that one of the most important causes of obesity is dietary factors (45), and hypertension is defined as a “lifestyle disease,” and daily behavioral habits are the risk factors for hypertension, including smoking, alcohol, diet, and physical activity (46), and obesity is one of the most important risk factors for hypertension (47, 48), and up to two-thirds of hypertension cases are related to overweight (49, 50). Therefore, one of the important reasons for obesity and hypertension is caused by their own unhealthy dietary choices. This type of patients may have their own dietary preferences, which is less likely to be affected by the boosting strategy.

**Table 5:** The expected intervention effects of three types of nudging strategies on different consumers (G & H).

Group	Variables		Categories	Providing decision information (I) OR (95% CI)	Changing decision structure (II) OR (95% CI)	Providing decision assistance (III) OR (95% CI)
G	Diseases	Obese	No (referent)	1 (referent)	1 (referent)	1 (referent)
			Yes	0.834 (0.711, 0.98)	0.941 (0.813, 1.089)	1.082 (0.932, 1.257)
		Hypertension	No (referent)	1 (referent)	1 (referent)	1 (referent)
			Yes	0.565 (0.512, 0.623)	1.068 (0.98, 1.164)	0.857 (0.779, 0.942)
		Diabetes	No (referent)	1 (referent)	1 (referent)	1 (referent)
			Yes	1.503 (1.364, 1.655)	1.032 (0.942, 1.13)	0.928 (0.838, 1.028)
		Hyperlipidemia	No (referent)	1 (referent)	1 (referent)	1 (referent)
			Yes	0.997 (0.911, 1.092)	1.126 (1.036, 1.224)	0.726 (0.662, 0.796)
		Gout	No (referent)	1 (referent)	1 (referent)	1 (referent)
			Yes	1.575 (1.415, 1.753)	1.709 (1.544, 1.891)	1.137 (1.016, 1.272)
		Tumors	No (referent)	1 (referent)	1 (referent)	1 (referent)
			Yes	1.186 (1.022, 1.376)	1.029 (0.901, 1.175)	1.287 (1.102, 1.503)
H	Dietary preference	Rice meals	Rarely purchase	1 (referent)	1 (referent)	1 (referent)
			Frequently purchase	0.534 (0.504, 0.567)	0.6 (0.569, 0.634)	0.586 (0.553, 0.621)
		Pizzas and hamburgers	Rarely purchase	1 (referent)	1 (referent)	1 (referent)
			Frequently purchase	0.739 (0.696, 0.785)	0.822 (0.777, 0.87)	0.801 (0.753, 0.852)
		Fast hot pot	Rarely purchase	1 (referent)	1 (referent)	1 (referent)
			Frequently purchase	0.97 (0.909, 1.035)	1.018 (0.959, 1.08)	0.933 (0.873, 0.998)
		Crayfish and barbecue	Rarely purchase	1 (referent)	1 (referent)	1 (referent)
			Frequently purchase	0.979 (0.918, 1.044)	0.99 (0.932, 1.051)	0.998 (0.934, 1.067)
		Fried chicken and fried skewers	Rarely purchase	1 (referent)	1 (referent)	1 (referent)
			Frequently purchase	0.771 (0.726, 0.82)	0.888 (0.84, 0.94)	0.816 (0.767, 0.869)
		Congee and gruel	Rarely purchase	1 (referent)	1 (referent)	1 (referent)
			Frequently purchase	0.929 (0.87, 0.993)	1.069 (1.006, 1.136)	0.944 (0.881, 1.011)
		Noodle set meal	Rarely purchase	1 (referent)	1 (referent)	1 (referent)
			Frequently purchase	0.978 (0.915, 1.044)	0.946 (0.889, 1.006)	0.945 (0.882, 1.013)
		Japanese and Korean cuisine	Rarely purchase	1 (referent)	1 (referent)	1 (referent)
			Frequently purchase	0.95 (0.822, 1.098)	1.129 (0.988, 1.291)	0.767 (0.661, 0.889)
		Western cuisine	Rarely purchase	1 (referent)	1 (referent)	1 (referent)
			Frequently purchase	1.116 (0.966, 1.29)	0.934 (0.812, 1.075)	0.776 (0.664, 0.907)
		Salad	Rarely purchase	1 (referent)	1 (referent)	1 (referent)
			Frequently purchase	0.647 (0.56, 0.748)	0.74 (0.649, 0.843)	0.688 (0.599, 0.789)
		Coffee	Rarely purchase	1 (referent)	1 (referent)	1 (referent)
			Frequently purchase	0.717 (0.658, 0.781)	0.802 (0.741, 0.867)	1.112 (1.021, 1.212)
		Fruits	Rarely purchase	1 (referent)	1 (referent)	1 (referent)
			Frequently purchase	0.759 (0.676, 0.851)	0.647 (0.579, 0.722)	0.856 (0.768, 0.954)
		Milk tea and desserts	Rarely purchase	1 (referent)	1 (referent)	1 (referent)
			Frequently purchase	0.74 (0.697, 0.786)	0.834 (0.788, 0.882)	0.845 (0.795, 0.899)

Consumers ‘personal dietary preferences are often one of the important factors in choosing foods (51), therefore, this paper analyzes the expected intervention effect of the boosting strategy according to the dietary preferences of the respondents (see Table 5). The results showed that respondents with fixed dietary preferences were basically not affected by the intervention of boosting strategies. For example, respondents who regularly ordered milk tea and desserts through the Internet platform were 0.740 of those who did not order milk tea and desserts frequently (95% CI 0.697 to 0.786), 0.834 (95% CI 0.788 to 0.882), 0.845 fold (95% CI 0.795 to 0.899). A large number of studies in behavioral economics and decision psychology point out that individual judgment and decision making process is not completely rational (52), especially in terms of diet choice, once people’s preference for unhealthy food is formed, it cannot be easily changed (11), and it will affect individual food choice for a long time. Therefore, respondents with fixed personal dietary preferences may ignore intervention strategies and may even develop emotional resistance, leading to rational behavior. Research Hypothesis 1 is valid, that is, the nudge strategy has a positive impact on the healthy eating behaviors of different types of respondents, and the impact varies based on eight different basic characteristics.

#### 4.2.2 Discuss the promotion strategies according to different categories

Previous studies have shown that the three major categories of facilitation strategies—information provision (Type I), structural change (Type II), and support provision (Type III)—are distinguished based on their different mechanisms of influence on decision-making systems. Accordingly, this paper also conducts a comparative analysis of how these three types of facilitation strategies affect respondents’ decisions. First, from the quantitative perspective (see Table 3), 54.53%, 54.04%, and 38.57% of respondents believe that their dietary choices are influenced by Type I, Type II, and Type III facilitation strategies, respectively. This suggests that the impact of support provision facilitation strategies may be less significant compared to the other two types. The reason might be that the support provision facilitation strategy designed in this study draws inspiration from Just and Gabrielyan (41), which involve direct verbal or written health reminders. Compared to the more “latent” facilitation strategies of the other two types, such direct reminders may trigger a natural resistance in consumers to some extent, leading to insufficient influence.

Through Table 4, this article found that respondents with older age, lower income and lower education are more susceptible to Type II boosting strategies, that is, changing the default sorting structure of online catering foods and increasing the visibility and availability of healthy foods can change the dietary choices of these respondents. Previous literature studies have also focused on the utility of this type of booster, for example, Cheung et al. (53) also found that increasing the visibility and availability of fruits (one of the structural booster strategies for changing decisions) can significantly increase the sales of fresh fruits. However, this paper further found that respondents aged 61–70 years old were 1.153 times more likely to be affected by the Type II boosting strategy than those aged 18–30 years old (95%CI 1.056 to 1.258). Previous studies have generally acknowledged that increasing the accessibility of healthy foods significantly improves the sales of healthy foods and is considered one of the key points of the boosting strategy, which is consistent with the research conclusions of this paper. On this basis, this paper also found that such boosting strategies are more influential among high-age, low-education and low-income consumer groups.

**Figure 2:**
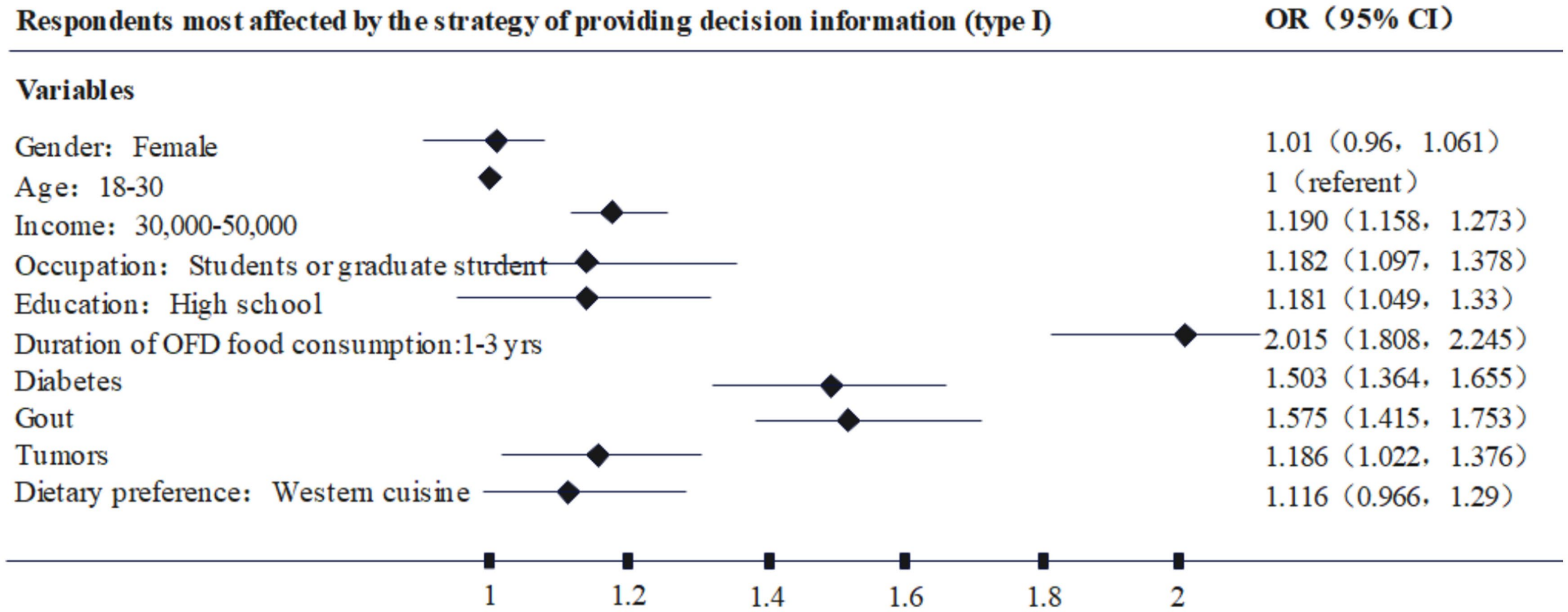
Respondents most affected by the strategy of providing decision information (type I).

**Figure 3:**
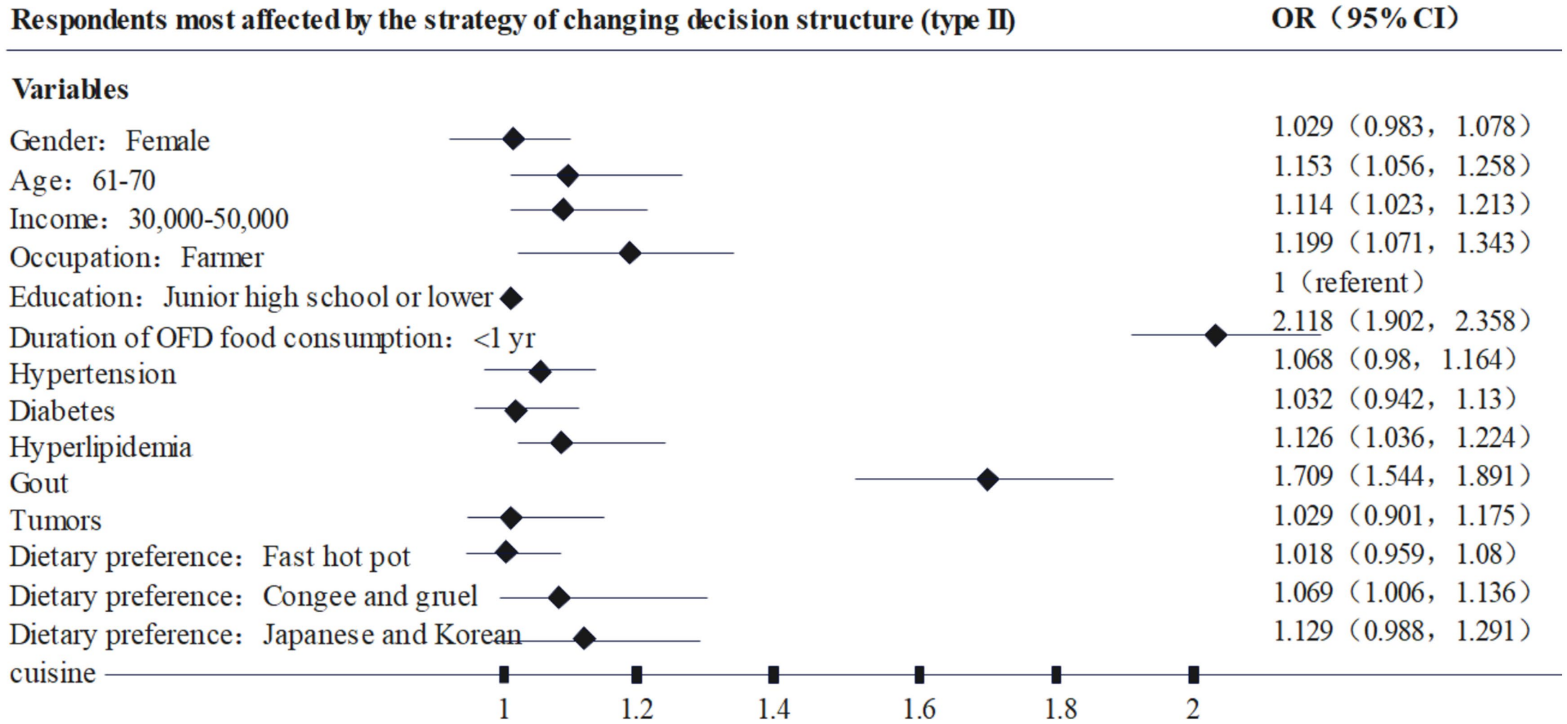
Respondents most affected by the strategy of changing decision structure (type II).

**Figure 4:**
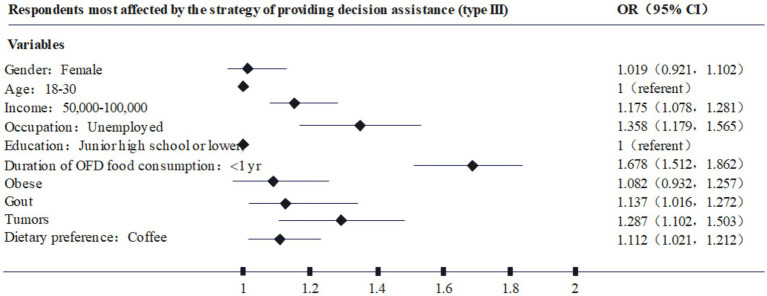
Respondents most affected by the strategy of providing decision assistance (type III).

Further, based on the different types of respondents, this paper plots Figures 2–4 based on the highest odds ratio (OR) of each type, summarizing the types of respondents who are most susceptible to type I, II, and III. nudging strategies. For example, Figure 2 shows that the characteristics of the respondents who are most likely to be affected by the Type I nudging strategy are: female, age 18–30, annual income of 3–50,000, occupation as student, high school education, online food consumption history of 1–3 years, diabetes, gout, cancer, and dietary preference for Western food. Through Figures 2–4, it can be found that the respondents who are susceptible to the impact of type I., II., and III. nudging strategies have some of the same characteristics, such as women, lower education levels, lower income consumers, and patients with chronic diseases are more susceptible to the intervention of healthy diet nudging strategies. However, there are differences in the impact of the three types of nudging strategies on respondents, for example, type I and type III nudging strategies are more likely to affect younger respondents, while type II is more influential on older respondents. At the same time, the three nudging strategies had different impacts on respondents with different occupations and dietary preferences. Therefore, research Hypothesis 2 holds, that is, different categories of nudge strategies have varying degrees of impact on the respondents’ healthy eating behaviors.

In the future, online platforms and nudging strategies may interact with wearable technologies for dietary behavior monitoring through the following approaches. First, it could be personalized dietary recommendations and reminders. When wearable devices detect that a user has engaged in a high level of physical activity on a given day, the online platform can send notification alerts to remind the consumer to supplement adequate protein and carbohydrates, and recommend corresponding foods or recipes—thereby guiding the consumer to make more rational dietary choices. Second, it could be self-monitoring and feedback of dietary behavior. After a consumer logs their daily diet on the online platform, if the wearable device detects abnormal fluctuations in the consumer’s blood glucose levels post-meal, the online platform can feed back this information to the consumer and prompt them to adjust their dietary structure (e.g., reducing intake of high-sugar foods). This leverages a feedback mechanism to nudge consumers toward improving their dietary behavior. Third, it could be long-term behavioral change and habit formation. By analyzing consumers’ long-term dietary behavior data and health data, online platforms can adopt behavior change techniques—such as goal-setting and gradual guidance—to help consumers establish healthy dietary habits. For instance, short-term and long-term dietary goals can be set for consumers (e.g., reducing intake of high-calorie foods by a certain amount per week). Through gradual guidance, consumers are assisted in modifying unhealthy dietary behaviors, ultimately fostering sustainable healthy dietary habits. This reflects the role of nudging strategies in guiding long-term behavioral change, as continuous support and guidance help users achieve sustainable improvements in dietary behavior.

## 5 Research conclusions and policy implications

Based on China scenario, this paper takes China market with large scale, many users and high penetration rate as a case study, collects 44,050 questionnaire data from consumers with online dining experience, and adopts three boosting strategies (provide decision information type, change decision structure type, provide decision assistance type) and respondents ‘self-reported willingness to accept the strategy as the main variables, using multiple logistic regression models to study the different expected effects of three boosting strategies on respondents with different consumer group characteristics, and summarize the different expected intervention effects of three boosting strategies. The results showed that research hypothesis 1 is valid: nudge strategies have a positive impact on the healthy eating behaviors of different types of respondents, and their influencing power varies based on eight distinct basic characteristics. Specifically, this is reflected in the fact that women, people with lower educational backgrounds, consumer groups with lower incomes, and chronic disease patients are more susceptible to healthy eating nudge strategies, while the expected intervention effects of nudge strategies on respondents with different occupations and dietary preferences vary. Research hypothesis 2 holds: different categories of nudge strategies have varying degrees of impact on respondents’ healthy eating behaviors. Specifically, the influence of decision-aid nudge strategies may be less than that of decision-information-providing and decision-structure-altering nudge strategies, while decision-structure-altering nudge strategies are more impactful among the older age groups, people with lower educational backgrounds, and consumer groups with lower incomes.

The research findings of this paper may have certain reference value for future interventions in promoting healthy eating behaviors among online food consumers: The advantages of the facilitation strategy, such as its simplicity, low cost, and high implementation efficiency, are more prominent in the consumption model of online dining. As a third party connecting merchants and consumers, online platforms can fully leverage the benefits of information technology to innovatively design and effectively implement facilitation strategies. For example, they can highlight the visibility of healthy foods on menu pages; when consumers select or order food, small health reminders can be displayed with nutritional information and evaluative labels attached to various foods; after selection, they can also help calculate the total calorie content and unhealthy component levels of chosen foods, to remind consumers or suggest improvements in food combinations, etc. Moreover, how to develop personalized facilitation strategies for different types of online food consumers is equally noteworthy. Similar to traditional intervention strategies, the choice of facilitation strategies should be based on consumer segmentation. Strategists should first clearly define the characteristics of the target population for facilitation, further segmenting them according to these characteristics, and formulating different facilitation strategies based on the common features of each segment, thereby achieving twice the results with half the effort. In addition, on the premise that nudge strategies have already exerted positive impacts, their subsequent chain reactions will be far-reaching and multi-layered. At the consumer level, nudge strategies are conducive to the formation of consumers’ habits, long-term health benefits, the general improvement of health awareness, and the subtle changes in social norms. In terms of industries and markets, nudge strategies help to force food enterprises to reform and create new market opportunities. At the public policy and social level, they contribute to reducing the burden of public medical care and promoting health equity, among other things.

Of course, this study has certain limitations. For example, the conclusions of this study are based on the Chinese consumer survey, and whether these conclusions are common in other countries needs further verification. In addition, the current research on the effectiveness of boosting strategies for intervening in healthy eating behaviors is based on short-term research conducted in a small-scale practical environment. It is difficult to prove the long-term effectiveness of boosting strategies in real environments, and their long-term effectiveness remains to be verified.

## Data Availability

The original contributions presented in the study are included in the article/Supplementary material, further inquiries can be directed to the corresponding author.

## Footnotes

^1^The classification of catering types is provided by the two major online catering platforms within China, namely the official platforms of Meituan and ele.me. It conforms to the eating patterns of Chinese consumers and can objectively and fully reflect consumers’ purchasing preferences.

## References

[1] Statista. Online food delivery in the United States. Available online at: Available online at: https://www.statista.com/outlook/374/109/online-food-deliveryundefinednited-states (2024).

[2] Dai X Wu L Hu W. Nutritional quality and consumer health perception of online delivery food in the context of China. BMC Public Health. (2022) 22:21–32. doi: 10.1186/s12889-022-14593-9 36403009 PMC9675956

[3] WHO. Obesity and overweight, fact sheet N° 311. In. (2016). Geneva, Switzerland: World Health Organization.

[4] WHO, “The top 10 causes of death,” (2020). Available online at: https://www.who.int/news-room/fact-sheets/detail/the-top-10-causes-ofdeath

[5] Larentis AV Barbosa DNF Barbosa JLV. SALUS: a model for educational assistance in noncommunicable chronic diseases. IEEE Lat Am Trans. (2023) 21:360–6. doi: 10.1109/TLA.2023.10068839

[6] Merino J. Diabetes and blood pressure mediate the effect of obesity on cardiovascular disease. Int J Obes. (2021) 45:1629–30. doi: 10.1038/s41366-021-00838-x 34002032

[7] Menzies V Kelly D Yang G Starkweather A Lyon D. A systematic review of the association between fatigue and cognition in chronic noncommunicable diseases. Chronic Illn. (2021) 17:129–50. 30884965 10.1177/1742395319836472 PMC6832772

[8] Murray CJL Aravkin AY Zheng P Abbafati C Mabbas K Abbasi-Kangevari M. Global burden of 87 risk factors in 204 countries and territories, 1990–2019: a systematic analysis for the global burden of disease study 2019. Lancet. (2020) 396:1223–49. doi: 10.1016/S0140-6736(20)30752-2, PMID: 33069327 PMC7566194

[9] Lindstrom KN Tucker JA McVay M. Nudges and choice architecture to promote healthy food purchases in adults: a systematized review. Psychol Addict Behav. (2022) 37:87–103. doi: 10.1037/adb0000892, PMID: 36395010

[10] Wongprawmas R Andreani G Franchini C Biasini B Rosi A Dolgopolova I. Nudging Italian university students towards healthy and sustainable food choices: an online experiment. Food Qual Prefer. (2023) 111:104971. doi: 10.1016/j.foodqual.2023.104971

[11] Li J Yu T. Boost-based intervention strategy for healthy eating behavior. Adv Psychol Sci. (2020) 12:2052–63.

[12] Laiou E Rapti I Schwarzer R Fleig L Cianferotti L Ngo J. Review: nudge interventions to promote healthy diets and physical activity. Food Policy. (2021) 102:102–3.

[13] Horne BD Muhlestein JB Lappe DL Le VT May H Bair T. Patient decision support through electronic behavioral nudges: the improvement in medicine adherence through the implementation of personalized nudges: the ENCOURAGE randomized controlled trial. Am Heart J. (2022) 244:125–34. doi: 10.1016/j.ahj.2021.11.001, PMID: 34798073

[14] Vecchio R Cavallo C. Increasing healthy food choices through nudges: a systematic review. Food Qual Prefer. (2019) 78:103714. doi: 10.1016/j.foodqual.2019.05.014

[15] Hansen PG Schilling M Malthesen MS. Nudging healthy and sustainable food choices: three randomized controlled field experiments using a vegetablearian lunch-default as a normal signal. J Public Health (Oxf). (2021) 43:392–7. doi: 10.1093/pubmed/fdz154, PMID: 31786590 PMC8185453

[16] Chen HJ Weng SH Cheng YY Hsu Y Tsai M Chang Y. The application of traffic-light food labelling in a worksite canteen intervention in Taiwan. Public Health. (2017) 150:17–25. doi: 10.1016/j.puhe.2017.04.005, PMID: 28622567

[17] Thorndike A Gelsomin ED McCurley JL Levy DE. Calories purchased by hospital employees after implementation of a cafeteria traffic light–labeling and choice architecture program. JAMA Netw Open. (2019) 2:e196789. doi: 10.1001/jamanetworkopen.2019.6789, PMID: 31290988 PMC6624805

[18] Payne C Niculescu M. Can healthy checkout end-caps improve targeted fruit and vegetable purchases? Evidence from grocery and SNAP participant purchases. Food Policy. (2018) 79:318–23. doi: 10.1016/j.foodpol.2018.03.002, PMID: 41080625

[19] Otto AS Davis B Wakefield K Clarkson JJ Jeffrey Inman J. Consumer strategies to improve the efficacy of posted calorie information: How provincial norms nudge consumers to healthier consumption. J Consum Affairs. (2020) 54:311–41. doi: 10.1111/joca.12272

[20] Cesareo M Sorgente A Labra M Palestini P Sarcinelli B Rossetti M. The effectiveness of nudging interventions to promote healthy eating choices: a systematic review and an intervention among Italian university students. Appetite. (2022) 168:105662. doi: 10.1016/j.appet.2021.105662, PMID: 34474099

[21] Brar K Minaker LM. Geographic reach and nutritional quality of foods available from mobile online food delivery service applications: novel opportunities for retail food environment surveillance. BMC Public Health. (2021) 21:458. doi: 10.1186/s12889-021-10489-2 33676458 PMC7937239

[22] Wang H Abbas KM Abbasifard M. Global age-sex-specific fertility, mortality, healthy life expectancy (HALE), and population estimates in 204 countries and territories, 1950–2019: a comprehensive demographic analysis for the global burden of disease study 2019. Lancet. (2020) 396:1160–203. doi: 10.1016/S0140-6736(20)30977-6, PMID: 33069325 PMC7566045

[23] Robinson E Marty L Jones A White M Smith R Adams J. Will calorie labels for food and drink served outside the home improve public health? BMJ. (2021) 372:n40. doi: 10.1136/bmj.n40, PMID: 33472836

[24] Jia P Luo M Li Y Zheng J Luo J. Fast-food restaurant, unhealthy eating, and childhood obesity: a systematic review and meta-analysis. Obes Rev. (2021) 22:1–27. 10.1111/obr.12944 PMC7988557 31507064

[25] Dana LM Hart E McAleese A Bastable A Pettigrew S. Factors associated with ordering food via online meal ordering services. Public Health Nutr. (2021) 24:5704–9. doi: 10.1017/S1368980021001294, PMID: 33762026 PMC10195403

[26] Horta PM Matos JDP Mendes LL. Food promoted on an online food delivery platform in a Brazilian metropolis during the coronavirus disease (COVID-19) pandemic: a longitudinal analysis. Public Health Nutr. (2022) 25:1336–45. doi: 10.1017/S1368980022000489 PMC9043632 35232512

[27] Du Y Rong S Sun Y Liu B Wu Y Snetselaar L. Association between frequency of eating away-from-home meals and risk of all-cause and cause-specific mortality. J Acad Nutr Diet. (2021) 121:1741–1749.e1. doi: 10.1016/j.jand.2021.01.012, PMID: 33775622

[28] Swinburn B Kraak V Rutter H Vandevijvere S Lobstein T Sacks G. Strengthening of accountability systems to create healthy food environments and reduce global obesity. Lancet. (2015) 385:2534–45. doi: 10.1016/S0140-6736(14)61747-5, PMID: 25703108

[29] Adams J Mytton O White M Monsivais P. Why are some population interventions for diet and obesity more equitable and effective than others? The role of individual agency. PLoS Med. (2016) 13:e1002045. doi: 10.1371/journal.pmed.1002045, PMID: 27046234 PMC4821622

[30] Wilson AL Buckley E Buckley JD Bogomolova S. Nudging healthier food and beverage choices through salience and priming. Evidence from a systematic review. Food Qual Prefer. (2016) 51:47–64. doi: 10.1016/j.foodqual.2016.02.009

[31] Lamken D Wahnschafft S Eggers C. Public acceptance of default nudges to promote healthy and sustainable food choices. BMC Public Health. (2023) 23:2311. doi: 10.1186/s12889-023-17127-z 37993839 PMC10664270

[32] Valencic E Beckett E Collins CE Seljak BK Bucher T. Digital nudging in online grocery stores: a scoping review on current practices and gaps. Trends Food Sci Technol. (2023) 131:151–63. doi: 10.1016/j.tifs.2022.10.018

[33] Sunstein CR. Why nudge? The politics of libertarian paternalism. New Haven, CT: Yale University Press (2014).

[34] Hansen PG Jespersen AM. Nudge and the manipulation of choice: a framework for the responsible use of the nudge approach to behaviour change in public policy. Eur J Risk Regul. (2013) 4:3–28. doi: 10.2307/24323381

[35] Johnson EJ Shu SB Dellaert BGC Fox C Goldstein DG Häubl G. Beyond nudges: tools of a choice architecture. Mark Lett. (2012) 23:487–504. doi: 10.1007/s11002-012-9186-1

[36] Cadario R Chandon P. Which healthy eating nudges work best? A meta-analysis of field experiments. Market Sci. (2020) 39:465–486. doi: 10.1287/mksc.2018.1128

[37] Münscher R Vetter M Scheuerle T. A review and taxonomy of choice architecture techniques. J Behav Decis Making. (2016) 29:511–24. doi: 10.1002/bdm.1897

[38] Bang HM Shu SB Weber EU. The role of perceived effectivity on the acceptance of choice architecture. *Behavioural*. Public Policy. (2020) 4:50–70.

[39] Loibl C Sunstein CR Rauber J Reisch LA. Which europeans like nudges? Approval and controversy in four european countries. J Consum Aff. (2018) 52:655–88. doi: 10.1111/joca.12181

[40] Carroll KA Samek A Zepeda L. Food bundling as a health nudge: investigating consumer fruit and vegetable selection using behavioral economics. Appetite. (2018) 121:237–48. doi: 10.1016/j.appet.2017.11.082, PMID: 29137968

[41] Just DR Gabrielyan G. Influencing the food choices of SNAP consumers: lessons from economics, psychology and marketing. Food Policy. (2018) 79:309–17. doi: 10.1016/j.foodpol.2018.03.003

[42] Bates S Reeve B Trevena H. A narrative review of online food delivery in Australia: challenges and opportunities for public health nutrition policy. Public Health Nutr. (2020) 1:1–11. doi: 10.1017/S1368980020000701 PMC7613985 32515719

[43] Eu EZR Sameeha MJ. Consumers’ perceptions of healthy food availability in online food delivery applications (OFD apps) and its association with food choices among public university students in Malaysia. Front Nutr. (2021) 8:674427. doi: 10.3389/fnut.2021.674427, PMID: 34497818 PMC8419248

[44] Mazza MC Dynan L Siegel RM Tucker AL. Nudging healthier choices in a hospital cafeteria: results from a field study. Health Promot Pract. (2018) 19:925–34. doi: 10.1177/1524839917740119, PMID: 29169270

[45] Masood B Moorthy M. Causes of obesity: a review. Clin Med. (2023) 23:284–91. doi: 10.7861/clinmed.2023-0168, PMID: 37524429 PMC10541056

[46] Colafella KMM Denton KM. Sex-specific differences in hypertension and associated cardiovascular disease. Nat Rev Nephrol. (2018) 14:185–201. doi: 10.1038/nrneph.2017.189, PMID: 29380817

[47] Re RN. Obesity-related hypertension. Ochsner J. (2009) 9:133–6. 21603428 PMC3096270

[48] Myette R Flynn JT. The ongoing impact of obesity on childhood hypertension. Pediatr Nephrol. (2024) 39:2337–46. doi: 10.1007/s00467-023-06263-8, PMID: 38189961

[49] Haslam DW James WP. Obesity. Lancet. (2005) 366:1197–209. 16198769 10.1016/S0140-6736(05)67483-1

[50] Fu Q. Sex differences in sympathetic activity in obesity and its related hypertension. Ann N Y Acad Sci. (2019) 1454:31–41. doi: 10.1111/nyas.14095, PMID: 31087350

[51] Jiang S Chen H Shan P Wang X. Efficacy of informational intervention on food waste: evidence from a randomized controlled trial. J Clean Prod. (2024) 443:141106. doi: 10.1016/j.jclepro.2024.141106

[52] Thaler RH. Misbehaving: The making of behavioral economics. New York/London: W. W. Norton & Company (2015).

[53] Cheung TTL Gillebaart M Kroese FM Marchiori D Fennis BM De Ridder DTD. Cueing healthy alternatives for take-away: a field experiment on the effects of (disclosing) three nudges on food choices. BMC Public Health. (2019) 19:974. doi: 10.1186/s12889-019-7323-y 31331307 PMC6647265

[54] Centers for Disease Control and Prevention in the United States. (2020, June 30). CDC over weight and Obesity. https://www.cdc.gov/obesity/index.html

