# Supplementary material for: Research on the expected utility of the nudge-based intervention strategy for healthy eating behavior in online catering
Source: Front Public Health. 2025 Oct 28;13:1644713. doi: 10.3389/fpubh.2025.1644713 (PMC12602542; doi:10.3389/fpubh.2025.1644713)
Supplement: Supplementary file 1 [file Data_Sheet_1.PDF]

Online Delivery Food Questionnaire

The first part of the basic situation of the interviewees

1. Your gender: (1) male; (2) female. （ ）

2. Your age: (1) 18-30 years old; (2) 31-45 years old; (3) 46-60 years old; (4) 61-70 years old (under 18 years old and over 70 years old, investigation is suspended). （ ）

3. Your marital status: (1) unmarried; (2) married. （ ）

4. Your education: (1) junior high school or below; (2) high school (including secondary vocational); (3) junior college; (4) undergraduate; (5) postgraduate and above. （ ）

5. Your annual income is: (1) 30,000 yuan and below; (2) 30,000-50,000 yuan; (3) 50,000-150,000 yuan; (4) 100,000-150,000 yuan; (5) 150,000-200,000 yuan; (6) 200,000 yuan or more. （ ）

6. Your occupation: (1) civil servant; (2) enterprise employee; (3) farmer; (4) institution employee; (5) freelancer; (6) retiree; (7) unemployed; (8) student; (9) others. （ ）

7. Your current place of residence is: (1) urban (including towns); (2) rural. （ ）

8. The population of your family (referring to the number of people living together in daily life): (1) 1 person; (2) 2 people; (3) 3 people; (4) 4 people; (5) 5 people or more. （ ）

9. Do you have children under the age of 12 in your family: (1) Yes; (2) No. （ ）

10. Your current height is______ and your weight is______ .

11. Based on what you know so far, do you have the following diseases (multiple choice, unlimited number): (1) hypertension; (2) diabetes; (3) hyperlipidemia; (4) gout; (5) tumor; (6) none of the above diseases. （ ）

12. Your place of residence is: 【 】 province (or autonomous region, municipality directly under the Central Government), 【 】 city (including autonomous prefectures in ethnic minority areas, etc.) 【 】 district (county). For example: Xinwu District, Wuxi City, Jiangsu Province;

The second part is to buy online delivery catering food

Note: The online delivery food referred to in this survey refers to the delivery food that can be used directly after purchase, not semi-finished products (pre-made dishes), and does not require reprocessing.

1. How ordering takeout affects your economic cost of living (regardless of time cost) compared to home cooking: (1) increases the cost of living; (2) reduces the cost of living; (3) is unclear. （ ）

2. Please estimate how often you order delivery food by yourself: (1) hardly buy it; (2) 1-2 times a week; (3) 3-4 times a week; (4) 5-6 times a week; (5) more than 6 times a week. （ ）

3. Please estimate how long you have been in the habit of ordering online delivery food: (1) almost no purchase; (2) less than 1 year; (3) 1-3 years; (4) 3-5 years; (5) 5 years and above. （ ）

4. If you have eaten online delivery food for 5 years or more, do you have the following physical changes (respondents who have not eaten for 5 years do not choose. Respondents over 5 years can choose from multiple, unlimited numbers): (1) increased weight; (2) increased blood lipids; (3) increased blood sugar; (4) increased blood pressure; (5) malnutrition; (6) gastrointestinal discomfort; (7) others; (8) no change. （ ）

5. What types of online delivery food do you often buy (multiple choices, limited to 3 items): (1) meal set meals; (2) hamburger pizzas; (3) hot fishing; (4) lobster barbecue; (5) fried chicken skewers; (6) porridge and noodles; (7) pasta set meals; (8) Japanese and Korean food; (9) western food takeaway; (10) light salads; (11) coffee; (12) fruit; (13) milk tea desserts. （ ）

6. Are you satisfied with the current safety status of online delivery food: (1) satisfied; (2) dissatisfied; (3) not sure. （ ）

7. Compared with before the epidemic, you feel that the current online delivery food safety situation: (1) has improved; (2) has not changed significantly; (3) has deteriorated. （ ）

8. Are you satisfied with the current government's ability to supervise the safety of online delivery food: (1) satisfied; (2) dissatisfied; (3) unclear. （ ）

9. Human health has a great relationship with the variety of food (dietary structure) of three meals a day. In your opinion, whether long-term consumption of online delivery food can meet people's daily health and nutritional needs: (1) it can be met; (2) it cannot be met; (3) it is uncertain. （ ）

10. If you feel that eating online take-out food for a long time is not good for your health, why do you often eat online take-out food (multiple choices, limited to 3 items): (1) work fast to save time; (2) have developed living habits; (3) the work unit does not have a canteen to meet the need for lunch; (4) need to continue working late or at night after get off work, eating take-out food can increase rest or save time to continue working; (5) Evening work requires additional supper. （ ）

11. Which of the following do you think will help to promote the governance of online delivery food safety (multiple choices, unlimited number): (1) The government has increased the supervision of online delivery food safety and illegal penalties; (2) clarified and strictly enforced the access conditions for takeaway operators; (3) established social institutions to participate in online delivery food safety supervision; and (4) consumers participate in online delivery food safety complaints and reports. （ ）

12. In your opinion, the main nutritional problems of online delivery food are (multiple choices, limited to 3 items): (1) the dishes are too oily; (2) the dishes are salty; (3) the seasonings such as monosodium glutamate are too much; (4) the dishes are high in energy; (5) the raw materials of the dishes are not fresh; (6) the vegetables in one meal are too little; and

(7) the staple food in one meal is too little. （ ）

13. Do you understand the Dietary Guidelines for Chinese Residents (2022), and if so, through which channels (multiple choices are available, limited to 3 items): (1) do not understand; (2) hospitals or clinics; (3) government departments; (4) non-profit organizations; (5) media software; and (6) others. （ ）

14. When ordering takeaway, if there is a food nutrition prompt label on the webpage, will it be helpful for you to buy healthy food: (1) helpful; (2) not helpful; (3) not necessarily. （ ）

15. When ordering online delivery food, will it make it easier for you to buy them if healthier catering foods are at the forefront: (1) it will be easier to buy them; (2) ignore them and still choose what you want; (3) not necessarily. （ ）

16. When ordering online delivery food t, if the question "What kind of vegetables do you want for lunch" appears on the webpage, will it affect your food choices: (1) Yes; (2) No; (3) Not necessarily. （ ）

17. If the reminder "You don't seem to have eaten very healthy today" appears on the webpage during the settlement of the order, will you be asked to go back and re-select: (1) Yes; (2) No; (3) Not necessarily. （ ）

Thank you again for taking the time to complete this questionnaire!
